# Microbial exchange at the wildlife-livestock interface: insights into microbial composition, antimicrobial resistance and virulence factor gene dynamics in grassland ecosystems

**DOI:** 10.1186/s42523-025-00448-2

**Published:** 2025-08-06

**Authors:** Lea Kauer, Panagiotis Sapountzis, Christian Imholt, Christian Berens, Ralph Kuehn

**Affiliations:** 1 Molecular Zoology, Department of Zoology, TUM School of Life Sciences, Technical University of Munich, Munich, Germany; 2 Medis 0454, INRAE-UCA, Centre INRAE Auvergne-Rhône-Alpes, Site de Theix, 63122 France; 3 Julius Kühn-Institute, Federal Research Centre for Cultivated Plants, Institute for Epidemiology and Pathogen Diagnostics, Rodent Research, Münster, Germany; 4 Friedrich-Loeffler-Institut, Institute of Molecular Pathogenesis, Jena, Germany; 5 Department of Fish, Wildlife and Conservation Ecology, New Mexico State University, Las Cruces, NM USA

**Keywords:** Metagenome assembled genomes, Virulence factors, Antimicrobial resistance genes, Grassland, Wildlife, Livestock, Common voles, Cattle, Sheep

## Abstract

The transmission of antimicrobial resistance genes (ARGs) and virulence factors (VFs) between wildlife and livestock is an emerging concern for animal and human health, especially in shared ecosystems. ARGs enhance bacterial survival against antibiotics, while VFs contribute to infection processes, and the microbiome composition influences host health. Understanding microbial exchange at the wildlife-livestock interface is essential for assessing risks to both animal and human health. This study addresses the gap in knowledge by investigating the microbial composition, ARGs, and VFs in fecal matter from livestock (*Bos taurus*, *Ovis aries*) and wildlife (*Microtus arvalis*) cohabiting grassland pastures. Sampling was conducted within the DFG Biodiversity Exploratories, which provides valuable and extensive long-term ecological datasets and enables the study of diverse environmental parameters. Using metagenomic sequencing and 16 S rRNA amplicon analysis, we compared bacterial diversity, antimicrobial resistance profiles, and virulence gene presence across the three host species. Metagenomic analysis revealed host-specific differences in bacterial community composition. Livestock samples exhibited higher microbial diversity than those from *M. arvalis*, likely due to greater environmental exposure and management practices. The most common VFs in livestock were associated with immune modulation, whereas motility-related VFs were prevalent in *M. arvalis*. ARG profiles differed among hosts, suggesting rare events rather due to environmental acquisition than direct transmission between the hosts. The limited numbers of ARGs and VFs shared between the species indicate that horizontal gene transfer events between wildlife and livestock are infrequent. Notably, *M. arvalis* harbored diverse ARGs, including resistance to tetracycline and vancomycin, which were likely acquired from the environment rather than from direct livestock contact. These findings highlight the significant role of environmental reservoirs in shaping microbial communities and the spread of resistance. This research underscores the need for enhanced surveillance and ecosystem management strategies to mitigate the risk associated with antimicrobial resistance and the potential impacts on both animal and human health.

**Supplementary Information:**

The online version contains supplementary material available at 10.1186/s42523-025-00448-2.

## Background

The transmission of bacteria, antimicrobial resistance genes (ARGs), and virulence factors (VFs), between wildlife and livestock has been increasingly recognized as a critical factor influencing disease ecology and antimicrobial resistance evolution [1–3]. ARGs contribute to the resilience of bacterial pathogens against antibiotic therapy, threatening the efficacy of essential treatments [4], VFs play a central role in the infection process through, e.g., host colonization and pathogen survival [5], and the composition of the microbiome significantly impacts host physiology, e.g., in contributing to health maintenance, disease susceptibility modulation and immune responses [6]. Antimicrobial resistance determinants and virulence factors, as well as the composition of the diverse microbiomes, thus, have profound relevance for human and animal health.

Inhabitating the same environment can lead to adaptation of the microbial composition among individuals or between individuals and their environment [7–9], indicating that both environment and interactions between individuals can play significant roles as external factors influencing the composition of the gut microbiota [10]. Transmission of ARGs, VFs and microbes between wildlife and livestock can occur, for example, through direct or indirect contact via, e.g., common water and food sources [2]. A recent publication by Wiethoelter, Beltrán-Alcrudo et al. [11] found that birds, carnivores, artiodactyls, rodents and bats, representing wildlife, and poultry, cattle, small ruminants, pigs and equines, representing livestock, accounted for 74% of the wildlife-livestock interface, underscoring the importance of these animal groups for the transmission and ecology of infectious diseases.

Additionally, anthropogenic land-use changes, e.g., shifts in farming practices, land-use intensification, or deforestation, influence epidemiological patterns [12, 13]. Intensive grazing systems and habitat overlap between wildlife and livestock create hotspots for microbial exchange, necessitating detailed investigation.

In this pilot study, we conducted metagenomic sequencing of fecal matter from *Microtus arvalis* (common voles) and either *Bos taurus* (cattle) or *Ovis aries* (sheep) inhabitating the same pastures at the same time. By comparing metagenomic data from these livestock and wildlife species, we aim to identify patterns of microbial overlap and divergence, assess the extent of shared ARGs and VFs, and elucidate potential transmission pathways at the wildlife-livestock interface. This research seeks to provide a basis for understanding how microbial communities and resistance elements circulate in multi-host systems. This knowledge is critical for understanding the risks posed by bacteria, ARGs and VFs, both for animal and human health, at the wildlife-livestock interface and for developing strategies to mitigate them.

## Results

### Bacterial composition of pooled vs. individual *M. arvalis* samples based on 16 S rRNA sequence data

**Fig. 1:**
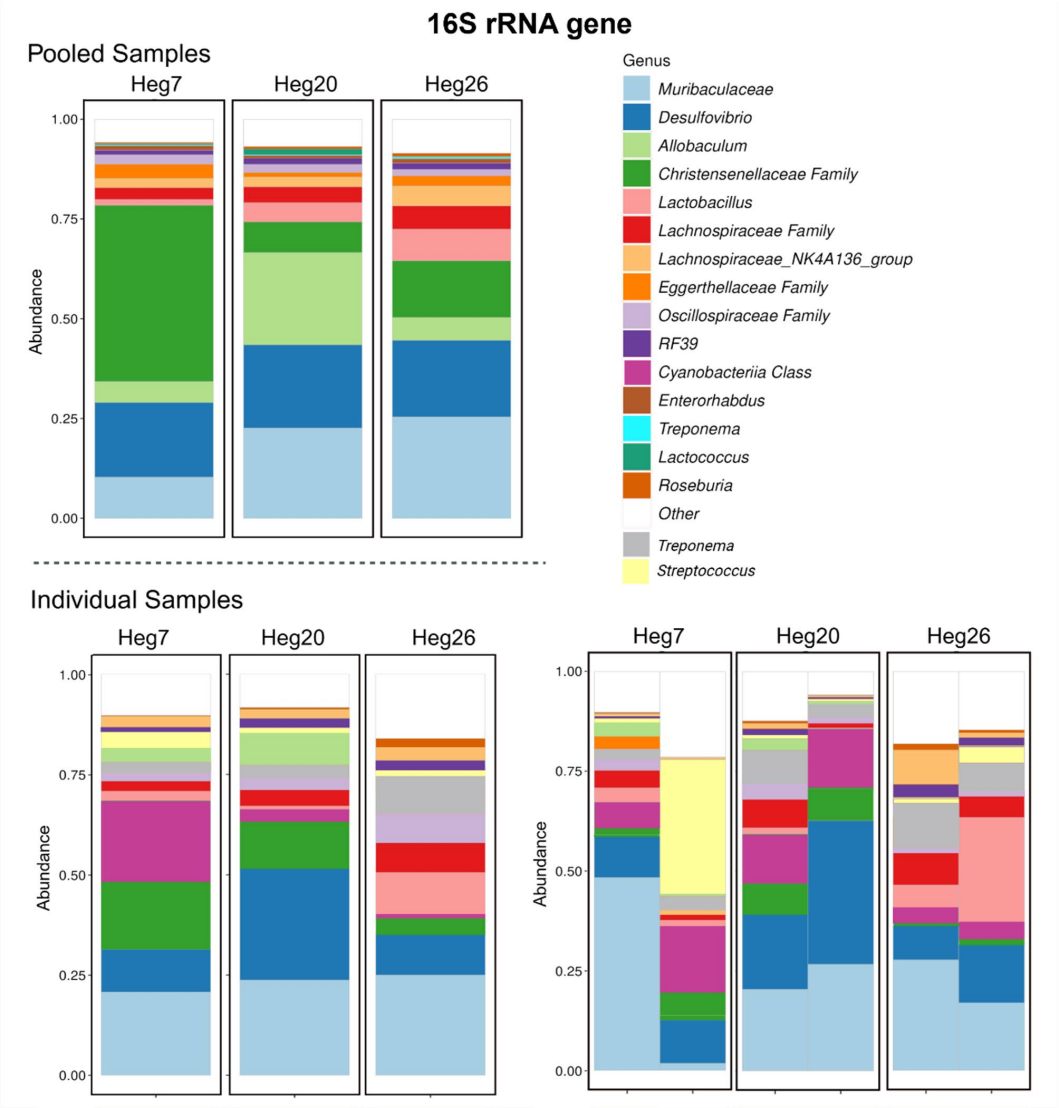
Taxonomic composition of pooled and individual samples of *M. arvalis* from the experimental plots. The individual samples on the bottom left represent pooling of the respective two individual samples from each plot on the right

**Fig. 2:**
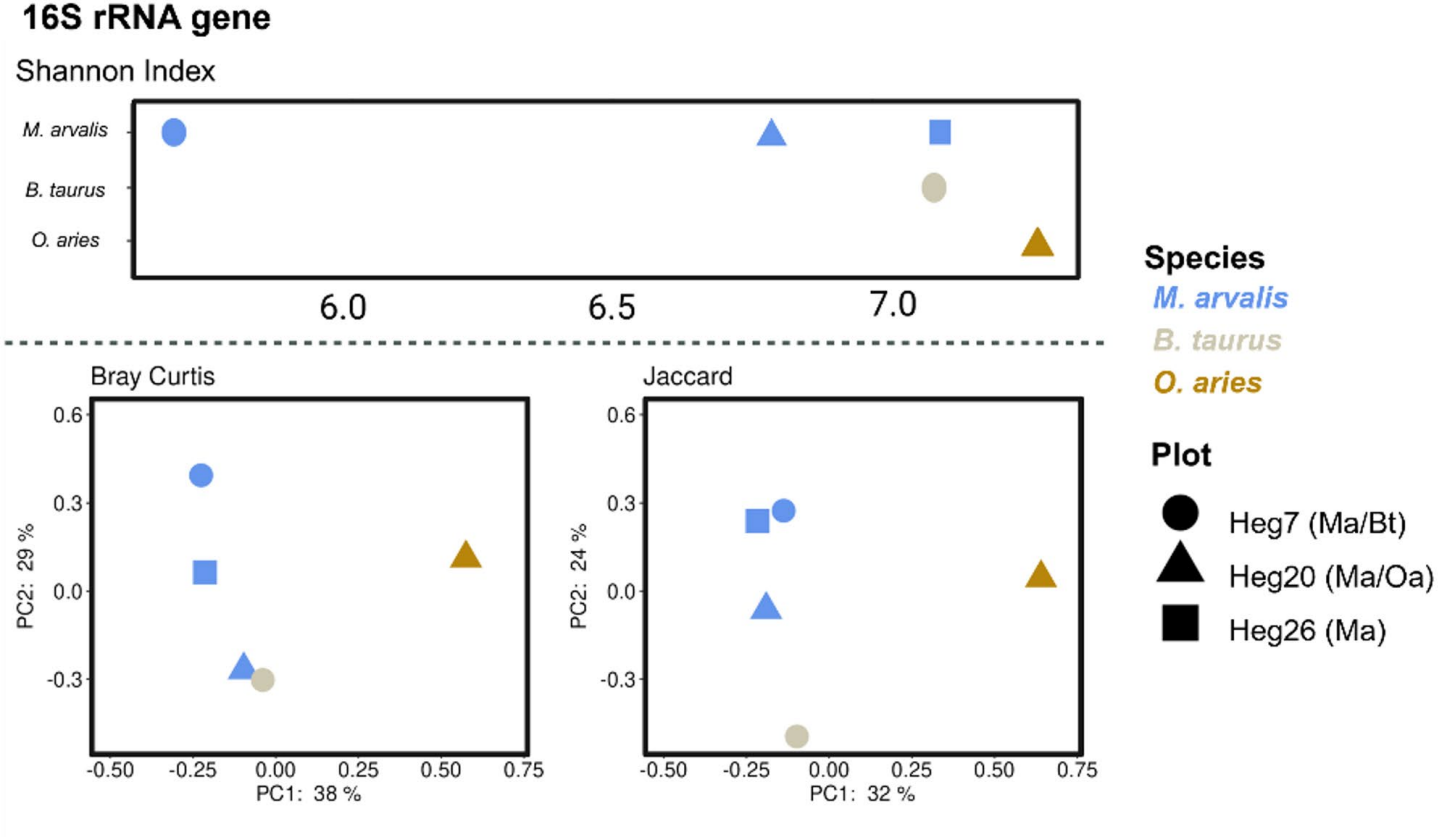
Alpha diversity (Shannon Index) and PCoA of beta diversity indices (Bray Curtis and Jaccard dissimilarity) for the 16 S rRNA sequencing data of the pooled *B. taurus*, *M. arvalis* and *O. aries* samples

Fecal matter of cattle and sheep origin collected at the respective grazing plot could not be assigned to individual animals and was stored and analyzed as a pooled sample. We, therefore, treated the murine samples similarly, also to compensate for the large degree of individual variation in microbiota samples and to ensure that sufficient bacterial content was present in the rodent samples for analysis. The most abundant genera were similar between the pooled and the pooled individual *M. arvalis* samples (see Table 1 and Supplementary material tables S1 and S2). Figure 1 shows the taxonomic composition of the pooled (top) and the two individual samples (bottom) from each plot at the genus level. The two individual samples were combined to generate the “pooled” barplots on the bottom left. The pooled samples effectively represent the pooled individual samples, with the former showing more *Allobaculum* spp. and the latter showing more bacteria belonging to class *Cyanobacteria*. The differences between the two individual samples from the Heg7 (Ma/Bt) plot are high. Therefore, no inference was made to the pooled samples. Overall, the individual sample variation is high (Figs. 1 and 2). The most abundant genera present in the pooled *M. arvalis* samples match those identified for the month of September in the study by Kauer, Imholt [14].

**Table 1:** Top five most abundant bacterial groups, based on 16 S rRNA sequencing within the pooled and individual *M. arvalis* samples pooled across all three plots

Host species and sample group	Subphylum taxonomic unit	Abundance [%]
*M. arvalis * *- individual samples*	*Muribaculaceae*	27
	*Desulfovibrio*	19
	*Lactobacillus*	7
	*Allobaculum*	4
	*RF39*	3
*M. arvalis–* *pooled samples*	*Desulfovibrio*	21
	*Muribaculaceae*	21
	*Allobaculum*	12
	*Lactobacillus*	5
	*Lachnospiraceae_NK4A136_group*	4

### Bacterial composition of pooled samples– comparison of data from 16 S rRNA sequencing vs. whole genome sequencing

#### Data from 16 S rRNA sequencing

A total of 1,347,662 paired-end reads with a median of 81,206 reads per sample was obtained by 16 S rRNA gene sequencing after quality control, chimera removal, and prevalence filtering. For all three host species, taxonomic classification assigned a majority of the reads to the Phylum *Firmicutes*, followed by *Bacteroidota*, and *Desulfobacterota* (Table 2). The top three phyla were present in all samples, the remaining two in two of three samples and none was unique to a host. At the subphylum level, the differences in the top five taxonomic units were more pronounced. Here, among the most abundant bacteria, only *Desulfovibrio* and *Allobaculum* were represented in all hosts. *Muribaculaceae* were additionally present in the murine and bovine samples, and the remaining taxonomic units were unique to each host.

**Table 2:** Top five most abundant bacterial phyla and taxonomic units within host-specific 16 S rRNA datasets

Plot	Host species	Bacterial phylum	Abundance [%]	Bacterial species/family	Abundance [%]
Heg7 (Ma/Bt)	*B. taurus*	*Firmicutes*	32	*Desulfovibrio*	21
		*Bacterioidota*	24	*Muribaculaceae*	21
		*Desulfobacterota*	19	*Rodentibacter*	12
		*Proteobacteria*	18	*Allobaculum*	5
		*Actinobacteriota*	7	*Brevibacterium*	4
	*M. arvalis*	*Firmicutes*	65	*Christensenellaceae*	44
		*Desulfobacterota*	19	*Desulfovibrionaceae*	19
		*Bacteroidota*	11	*Muribaculaceae*	10
		*Actinobacteriota*	6	*Lachnospiraceae*	7
		*Proteobacteria*	1	*Erysipelotrichaceae*	5
Heg26 (Ma)		*Firmicutes*	49	*Muribaculaceae*	26
		*Bacteroidota*	56	*Desulfovibrionaceae*	19
		*Desulfobacterota*	19	*Christensenellaceae*	15
		*Actinobacteriota*	5	*Lachnospiraceae*	13
		*Spirochaetota*	1	*Lactobacillaceae*	8
Heg20 (Ma/Oa)		*Firmicutes*	23	*Erysipelotrichaceae*	23
		*Bacteroidota*	23	*Muribaculaceae*	23
		*Desulfobacterota*	21	*Desulfovibrionaceae*	21
		*Actinobacteriota*	2	*Lachnospiraceae*	9
		*Proteobacteria*	1	*Christensenellaceae*	8
	*O. aries*	*Firmicutes*	75	*Christensenellaceae_R-7_group*	21
		*Bacterioidota*	16	*UCG-005*	17
		*Desulfobacterota*	6	*Allobaculum*	15
		*Spirochaetota*	1	*Desulfovibrio*	13
		*Proteobacteria*	1	*NK4A214_group*	5

The *O. aries* sample had the highest alpha diversity, followed by the *M. arvalis* sample MP26 from the control plot and the *B. taurus* sample. The *M. arvalis* sample MP7 from the cattle-grazed plot had the lowest alpha diversity. Beta diversity measures Bray-Curtis and Jaccard dissimilarity showed slightly different clusters, with the Jaccard index clustering the *M. arvalis* pooled samples closer together than the Bray-Curtis distance (Fig. 2). In both cases, the *M. arvalis* samples clustered closer together than the corresponding vole/cattle and vole/sheep samples.

#### Data from whole genome sequencing

Whole genome sequencing (WGS) of the samples produced a total of 464,341,357 reads after quality filtering with a median of 92,887,652 paired-end reads per sample.

**Fig. 3:**
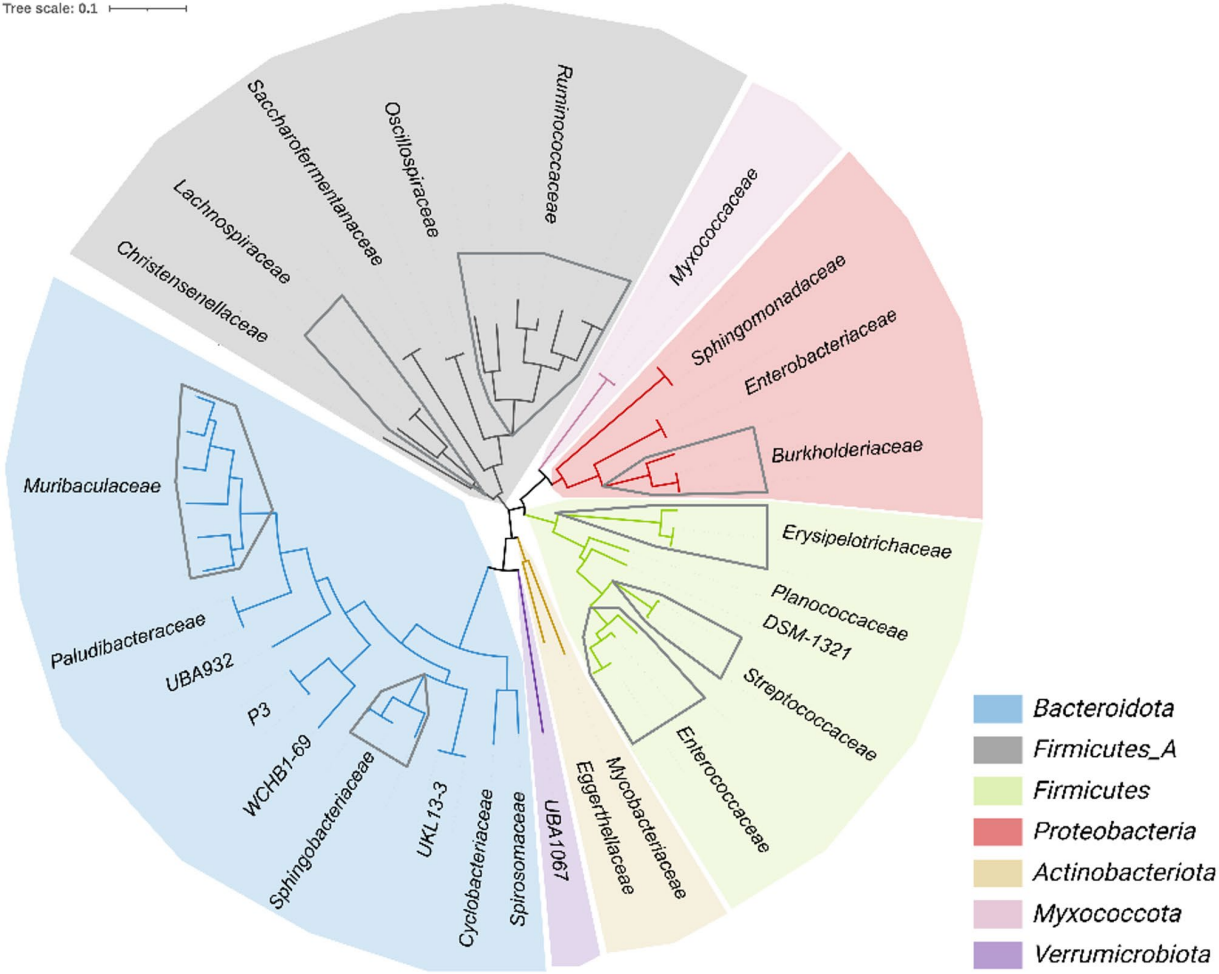
Phylogenetic diversity of metagenome-assembled genomes from *B. taurus*, *M. arvalis* and *O. aries* fecal microbiota

Construction of metagenome-assembled genomes with more than 80% completeness and less than 5% contamination resulted in a total of 58 MAGs. Taxonomic classification of these MAGs using GTDB-tk assigned most of the reads to the Phylum *Firmicutes* Syn. *Bacillota* (*Firmicutes_A*) for *B. taurus* and *O. aries*, followed by *Bacteroidota* and *Firmicutes*. The *M. arvalis* dataset was dominated by the Phylum *Bacteroidota*, followed by *Firmicutes_A* and *Proteobacteria* (Syn. *Pseudomonadota*). The majority of the reads belong to the bacterial species *Enterococccus_D casseliflavus*, *Allobaculum sp014803815*, and *Lactococcus garvieae* within all host species except for the *M. arvalis* dataset, where *Mycobacterium vaccae* dominated, followed by *Allobaculum sp014803815* and *Escherichia coli* (Table 3). The phylogenetic diversity of the MAGs is displayed in Fig. 3.

Classification of the reads using Kaiju is consistent with the classification of MAGs for *O. aries* regarding bacterial phyla. For the *M. arvalis* samples, Kaiju also found *Firmicutes* and *Bacteroidota* to be the most abundant phyla, but then *Actinobacteriota* followed. The Kaiju results for the *B. taurus* sample assigned most of the reads to the phylum *Proteobacteria*, followed by *Actinobaceriota* and *Bacteroidota*, which does not align with the finding of the MAGs, being dominated by *Firmicutes* and *Bacteroidota* (Table 3).

**Table 3:** Top five most abundant bacterial (MAG) phyla and species within host specific WGS datasets

Plot	Host species	Bacterial phylum	Abundance [%]	Bacterial Species	Abundance [%]
Heg7 (Ma/Bt)	*B. taurus*	*Firmicutes_A*	*49*	*Allobaculum sp014803815*	*79*
		*Bacteroidota*	*36*	*Enterococcus_D casseliflavus*	*12*
		*Firmicutes*	*14*	*Lactococcus garvieae*	*7*
		*Actinobacteriota*	*0.6*	*Mycobacterium vaccae*	*0.9*
		*Myxococcota*	*0.01*	*Enterococcus_B pernyi*	*0.3*
	*M. arvalis*	*Firmicutes_A*	*56*	*Allobaculum sp014803815*	*65*
		*Bacteroidota*	*37*	*Enterococcus_D casseliflavus*	*17*
		*Firmicutes*	*7*	*Lactococcus garvieae*	*16*
		*Actinobacteriota*	*1*	*Enterococcus_B*	*0.6*
		*Myxococcota*	*0.01*	*UBA1723 sp002392915*	*0.3*
Heg26 (Ma)		*Bacteroidota*	*39*	*Mycobacterium vaccae*	*88*
		*Actinobacteriota*	*31*	*Sphingobacterium alimentarium*	*10*
		*Proteobacteria*	*21*	*Enterococcus_D casseliflavus*	*1*
		*Myxococcota*	*8*	*Lysinibacillus boronitolerans*	*1*
		*Firmicutes*	*1*	*Allobaculum sp014803815*	*0.2*
Heg20 (Ma/Oa)		*Bacteroidota*	*37*	*Escherichia coli*	*31*
		*Proteobacteria*	*27*	*Firm-04 sp017533485*	*16*
		*Firmicutes_A*	*25*	*UBA1723 sp002392915*	*15*
		*Firmicutes*	*7*	*Saccharofermentans sp015069205*	*12*
		*Verrucomicrobiota*	*6*	*RUG11690 sp902771655*	*12*
	*O. aries*	*Firmicutes_A*	*37*	*Allobaculum sp014803815*	*72*
		*Bacteroidota*	*25*	*Lactococcus garvieae*	*25*
		*Firmicutes*	*23*	*Enterococcus_D casseliflavus*	*3*
		*Actinobacteriota*	*16*	*Enterococcus faecalis*	*0.4*
		*Proteobacteria*	*0.04*	*Escherichia coli*	*0.1*

**Fig. 4:**
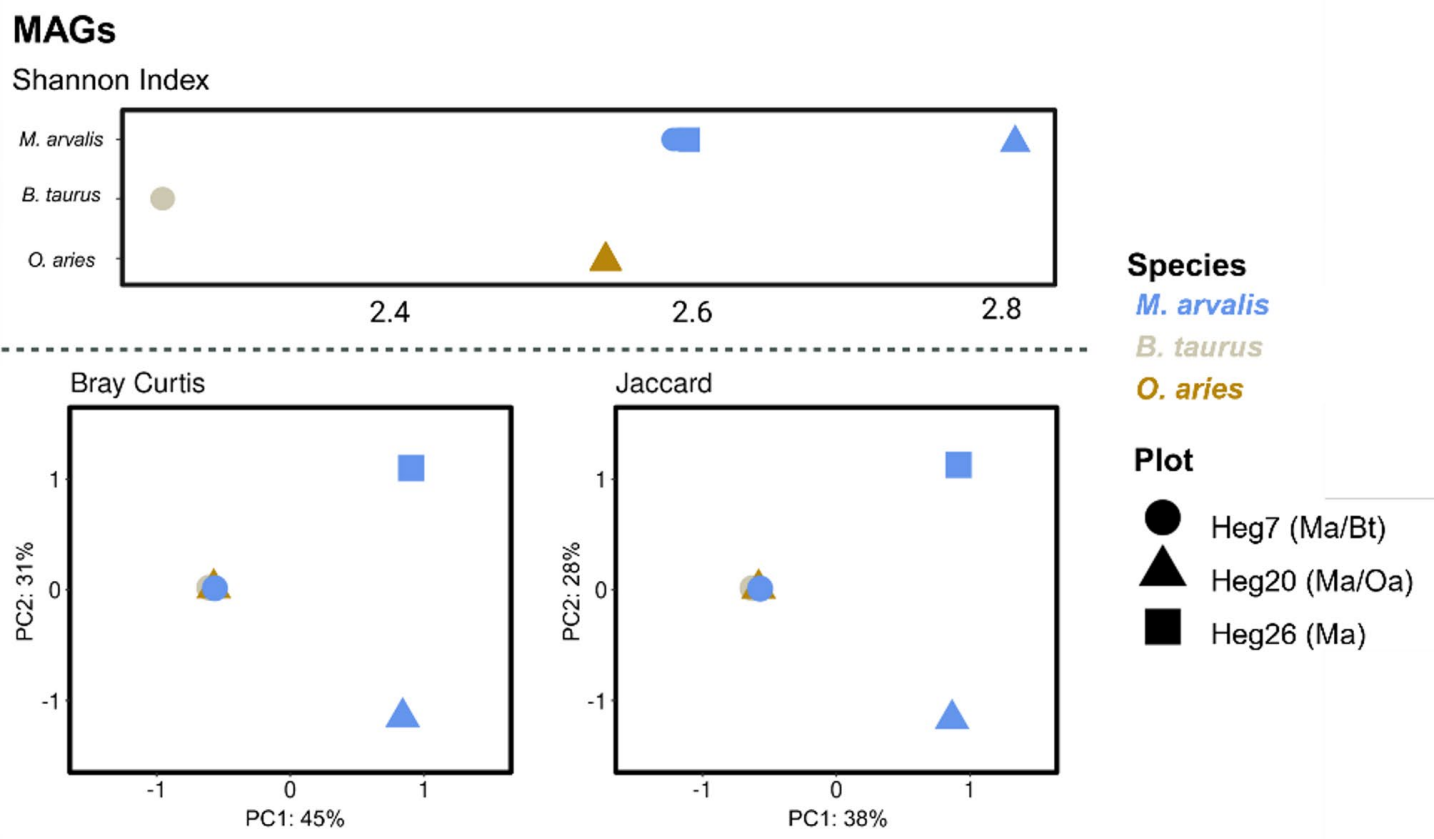
Alpha diversity (Shannon Index) and PCoA of beta diversity indices (Bray Crutis and Jaccard dissimilarity) for the MAGs of pooled *B. taurus*, *M. arvalis* and *O. aries* samples

Within the MAG dataset, the *M. arvalis* samples showed the highest alpha diversity (Shannon Index), followed by the *O. aries* sample, with *B. taurus* having the lowest alpha diversity. The beta diversity measures Bray Curtis and Jaccard dissimilarity indices showed similar results. The *B. taurus* and *O. aries* samples clustered together with the *M. arvalis* sample MP7, taken from the cattle pasture (Fig. 4).

### Comparison of the 16 S rRNA and WGS data

As expected, the composition of the bacterial communities differed in the 16 S rRNA, the Kaiju and the MAGs data. Even though *Firmicutes* and *Bacteroidota* made up most of the bacterial phyla within all three datasets, the composition of higher taxonomic ranks was barely comparable between the datasets. Comparison of phyla between 16S rRNA, Kaiju and MAGs showed that Kaiju analysis revealed a more pronounced phylum richness than the other two (Supplementary Material Figure S1). The 16 S rRNA dataset, for example, lacked the *Enterococcus* and *Escherichia* genera among the top 5 most abundant genera, as was seen in the MAGs composition. *Muribaculaceae* were highly abundant in both datasets, across all samples, except for the *O. aries* sample in the 16 S rRNA dataset. Other bacterial families, such as *Erysipelotrichaceae*, *Lachnospiraceae* and *Eggerthellaceae*, were also similarly abundant in both the 16 S rRNA and WGS datasets. Results of alpha and beta diversity derived from 16 S rRNA and MAGs differed. The *M. arvalis* samples had the highest alpha diversity in the MAG dataset, whereas in the 16 S rRNA dataset they not only had the lowest apha diversity but also a greater variability. Also, the beta diversity measures featured different clustering. While the *M. arvalis* samples appeared to cluster together in the 16 S rRNA dataset, the PCoA of the MAGs showed a different pattern, with the *B. taurus*, *O. aries* and the *M. arvalis* sample from plot Heg7 (Ma/Bt) clustering together. Overall, these findings highlight the importance of using multiple approaches for a more comprehensive understanding of microbial diversity.

### Resistome and virulence factors

#### Virulence factors

A total of 898 VF were detected in the dataset. The *B. taurus* samples held the most VFs (463), followed by the *O. aries* sample (289). The *M. arvalis* samples showed less, but similar numbers of VFs (Heg20 (Ma/Oa): 97, Heg7 (Ma/Bt): 82, Heg26 (Ma): 70). Despite the overall comparably high number of VFs detected in *B. taurus* and *M. arvalis* from the same plot (Heg7) (61%; *N* = 545), the two host species only share about 2.6% (*N* = 21) of the VFs. The same applies for the *O. aries* and *M. arvalis* samples from the same plot (Heg20) which harbor 43% (*N* = 383) of all VFs, but share only 2% (*N* = 19) of these VFs. Among the *M. arvalis* samples, 4.5–6.4% (*N* = 13–16) of the VFs are shared. Mapping of the reads on the VFdb database revealed that virulence-related genes associated with immune modulation, motility and adherence, which can promote host colonization and infection were found in all three hosts, albeit to different extents (Table 4). The livestock samples additionally featured VFs dealing with effector protein delivery and toxins, contributing to host cell damage, while, in contrast, the rodent samples contained factors important for biofilm formation and metabolism, also contributing to host colonization. The livestock samples showed a higher alpha diversity than the *M. arvalis* samples. The most abundant genes found (Type IV Pili, Flp Type IV Pili) are associated with adherence. Within all species, the VFs Flp type IV pili and *Streptococcal* plasmin receptor, both associated with adherence, were identified. Upon inspection of the virulence features of livestock and wildlife, a group of shared virulence factors was found, although the composition of the virulence factors shared between *M. arvalis* and *B. taurus* differed from the one shared between *M. arvalis* and *O. aries*. The *M. arvalis*– *B. taurus* VF set, which included intracellular survival mechanisms (ESX-1, ESX-3, Mce4, *PknG*, *PrrA/B*), immune evasion factors (PDIM, *KatG*), and metal acquisition systems (Heme uptake, Antigen 85, Zn + + metalloprotease), resembles virulence repertoires commonly seen in *Mycobacterium* spp. and other intracellular pathogens. The *M. arvalis*– *O. aries* VF set, enriched in adhesion factors (Type IV pili, *E. coli* fimbriae), toxins (CDT, Hemolysin), and secretion systems (T6SS, T7SS, Dot/Icm T4SS), aligns with a broader range of Gram-negative and Gram-positive bacterial virulence strategies, which are frequently associated with opportunistic or enteric pathogens.

**Table 4:** Top five most abundant VF groups and VF genes within the host specific WGS datasets

Plot	Host species	VF group	Abundance [%]	VF gene	Abundance [%]
Heg7 (Ma/Bt)	*B. taurus*	Motility	26	*Flagella*	26
		Effector delivery system	16	*Flp type IV pili*	20
		Adherence	14	*T6SS-III*	16
		Immune modulation	11	*LOS*	16
		Exotoxin	8	*MAM*	8
	*M. arvalis*	Motility	19	*Flagella*	19
		Biofilm	15	*LOS*	19
		Adherence	12	*Flp type IV pili*	17
		Immune_modulation	11	*Alginate*	15
		Exotoxin	8	*LOS*	10
Heg26 (Ma)		Immune modulation	18	*LAM*	33
		Nutritional/Metabolic factor	13	*Heme uptake*	21
		Effector delivery system	5	*GPL locus*	19
		Adherence	5	*Flp type IV pili*	12
		Motility	2	*Gsp*	5
Heg20 (Ma/Oa)		Motility	4	*HSI-1*	0.8
		Nutritional/Metabolic factor	2	*Alginate*	0.8
		Effector delivery system	1	*Streptococcal plasmin receptor*	0.7
		Adherence	1	*Enterobactin synthesis and transport*	0.6
		Biofilm	1	*Enterobactin*	0.6
	*O. aries*	Adherence	10	*Type IV pili*	19
		Immune modulation	7	*Flp type IV pili*	15
		Exotoxin	4	*LOS*	11
		Motility	3	*Capsule*	8
		Effector delivery system	3	*LOS*	7

**Fig. 5:**
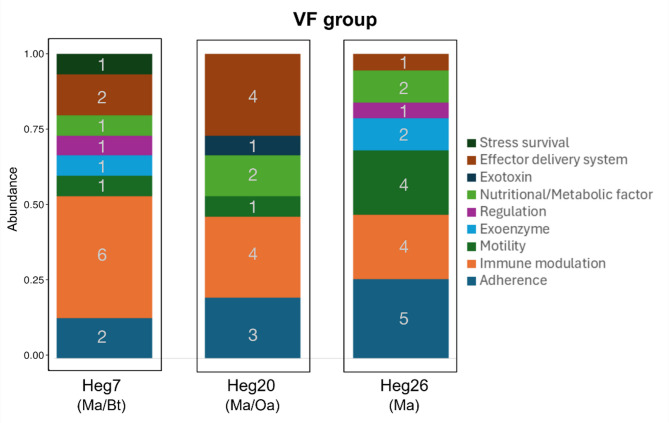
Composition of VF groups and number of VF genes unique for the respective plot but shared by *M. arvalis* and *B. taurus* (Heg7), by *M. arvalis *and *O. aries* (Heg20) or unique to the plot containing only *M. arvalis* (Heg26)

**Fig. 6:**
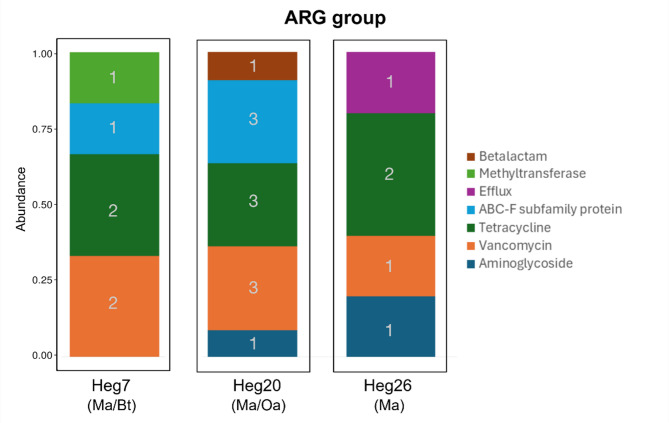
Composition of ARG groups and number of ARGs unique for the respective plot but shared by *M. arvalis* and *B. taurus* (Heg7), by *M. arvalis* and *O. aries* (Heg20) or unique to the plot containing only *M. arvalis* (Heg26)

The analysis of the MAGs with VFdb did not reveal any virulence-related genes within the MAG bins (Figs. 5 and 6).

### Antimicrobial resistance genes

A total of 99 ARG were detected in the dataset. The *O. aries* sample held the most ARGs (N=38), followed by the *B. taurus* sample (N=26) and the *M. arvalis* samples from Heg20 (Ma/Oa): 26, Heg7 (Ma/Bt): 21, and Heg26 (Ma): 15. Despite the overall comparably high numbers of ARGs detected in *O. aries* and *M. arvalis* from the same plot (Heg20) (65%; *N* = 64), the two host species only shared about 11% (*N* = 7) of the ARGs. The same applied to the *B. taurus* and *M. arvalis* samples from the same plot (Heg7), which harbored 47% (*N* = 47) of all ARGs, but shared only 6% (*N* = 3) of their ARGs. Among the *M. arvalis* samples, 9,6–14,5% (*N* = 6–9) of the ARGs were shared. Mapping of the reads using AMRFinderPlus revealed that most of the ARGs represented genes involved in vanconmycin resistance, over all host species, followed by genes associated with resistance to aminoglycosides, tetracyclines or lincosamides, depending on the host species (Table 5). The overall number of ARGs identified was low. *M. arvalis* showed the lowest alpha diversity, followed by *B. taurus* and *O. aries*, which had the highest alpha diversity with respect to the AMR gene composition. The comparison of ARGs between *M. arvalis* and livestock revealed only minimal overlap. A single plasmid-encoded gene encoding a β-lactamase was shared between *M. arvalis* and *B. taurus*, while four genes were shared between *M. arvalis* and *O. aries*, three of which were transporters (*mdtM* is endogenous in *E. coli*). Additionally, *tet* [40], a non-classical tetracycline resistance transporter gene, and a β-lactamase associated with Gram-negative bacteria were detected. Several resistance genes were detected in the *M. arvalis* samples but were absent in the livestock samples. They included tetracycline-resistance genes mediating ribosomal protection and vancomycin resistance genes. Several ARGs were found in both mouse and livestock samples, but not from the same sampling location, again including tetracycline-resistance genes mediating ribosomal protection, a vancomycin resistance gene, and a β-lactamases. *B. taurus* specific resistance genes were largely plasmid-encoded and acquired (*aad*, *blsADC*, *emr*, *floR*, *oqx*, *qep*, *sul2*, *tetG*, *tetX*, *tmex*), occurring in both Gram-negative and Gram-positive pathogens and commensals. In *O. aries*, numerous aminoglycoside resistance genes were identified. Endogenous *bla*_EC_ variants from *E. coli* were also detected, alongside acquired resistance determinants (*mefA*, *sat4*, *tetOQWNW*), as well as *cfrE*, *lnu*, *msrD*, and *emr/erm* genes, which are typically found in Gram-positive bacteria. Among the distinct *M. arvalis* populations, genes associated wuth aminoglycoside, tetracycline and vancomycin resistance (voles at Heg20 (Ma/Oa), Heg26 (Oa)) or lsa and *bla* genes (voles at Heg20 (Ma/Oa)) were detected, some of which are acquired resistance genes preferentially found in Gram-negatives. Tetracycline and vancomycin resistance genes were widespread, along with *lsa*, and in voles from the Heg07 (Ma/Bt) plot, *erm* and *fus* genes in addition, which are commonly associated with Gram-positive bacteria.

Examining the MAGs using AMRFinderPlus did not reveal any ARGs within the MAGs.

**Table 5:** Top five most abundant ARGs within the host specific WGS datasets

Plot	Host species	ARG group	Abundance [%]	ARG	Abundance [%]
Heg7 (Ma/Bt)	*B. taurus*	vancomycin	88	*vanR*	80
		tetracycline	5	*vanS*	4
		macrolide	2	*vanD*	2
		lincosamide	2	*tet(Q)*	2
		beta-lactam	1	*erm*	2
	*M. arvalis*	vancomycin	81	*vanR*	75
		tetracycline	9	*tet(32)*	8
		aminoglycoside	4	*vanS*	6
		beta-lactam	2	*aph*	4
		biocide	1	*bla*_TEM−157_	1
Heg26 (Ma)		aminoglycoside	37	*aadA6*	28
		efflux	17	*clpK*	12
		vancomycin	9	*vanR-Sc*	9
		macrolide	7	*aadA11*	8
		tetracycline	5	*sdeB*	8
Heg20 (Ma/Oa)		efflux	20	*air*	7
		acid	17	*asr*	7
		beta-lactam	7	*emrD*	7
		tetracycline	5	*fieF*	6
		lincosamide	2	*iss*	6
	*O. aries*	vancomycin	76	*vanR*	60
		lincosamide	9	*lsa(D)*	7
		tetracycline	6	*vanS*	10
		efflux	3	*tet(O)*	4
		beta-lactam	2	*vanH*	3

## Discussion

The results of the 16 S rRNA sequencing differ from the MAGs and Kaiju results, the latter two derived from WGS. While the composition is comparable at the phylum level, it diverges strongly at the lower taxonomic levels. This likely stems from the fact, that the 16 S rRNA gene metabarcoding introduces an additional amplification step to amplify the 16 S rDNA gene with a potential bias in sequence-dependent amplification efficiency. In contrast, the metagenomic approach relies on direct sequencing of the isolated DNA and, thus, taxa representation is to some extent dependent on sequencing depth [15], which was high in this study. Additionally, and importantly, the composition of the WGS dataset in our study is limited to 58 reliable MAGs created from the WGS dataset reads. The results of taxonomic classification of Kaiju, MAGs and 16 S rRNA gene reflect to a good extent these discrepancies. Kaiju and 16 S rRNA gene analyze raw reads, capturing a broader taxonomic diversity, while MAGs are limited by assembly efficiency, genome completeness, and binning accuracy. As a result, the taxonomic profiles derived from Kaiju and 16 S rRNA sequencing appear more similar, whereas MAG-based classification is constrained here to the relatively low number of 58 well-assembled genomes, leading to potential differences in community representation.

The bacterial composition of the *M. arvalis* samples is comparable to the composition found in other studies on *M. arvalis* [14, 16]. Our findings of *Firmicutes* and *Bacteroidota* making up a large majority of the *O. aries* and *B. taurus* microbiome are also in line with results from other studies [17–19]. At the family level, the results of the ruminant data differ from the above-mentioned studies, in which *Ruminococcaceae*, *Lachnospiraceae* and *Bacteroidaceae* dominated the bacterial microbiome. In contrast, we found *Desulfovibrionaceae*, *Muribaculaceae* and *Ruminocaccaceae* (*B. taurus*) and *Christensenellaceae*, *Oscillospiraceae* and *Lachnospiraceae* (*O. aries*) being the most abundant families. As Huebner, Martin [18] state, nutrition and management practices impact the microbiome of animals significantly. Therefore, the different husbandry conditions of the studies mentioned above compared to our study are probably responsible for the differences.

We were able to assemble 58 metagenome-assembled genomes (MAGs) from the feces analysed here. These are to our knowledge the first MAGs derived from *M. arvalis*.

Immune modulation, regulation, and adherence are the most common VFs in the livestock samples, whereas in *M. arvalis*, motility, immune regulation, and adherence are the most abundant VFs. Livestock samples, in this case from ruminants, showed significantly higher alpha diversity values than the samples from *M. arvalis*. This difference in diversity likely results from a combination of greater microbial exposure, antibiotic-driven selection, higher bacterial densities, and increased horizontal gene transfer in livestock than in *M. arvalis*. These findings align with studies performed in livestock which showed that management practices, diet and the use of antibiotics have the potential to promote the repertoire of VFs present [20, 21]. The VF profile shared between *M. arvalis* and *B. taurus* is characterized by genes associated with intracellular survival, immune evasion, and iron acquisition, exemplified by ESX-1, ESX-3, Mce4, *PknG*, *PrrA/B*, KatG, and Heme uptake, respectively. These factors are commonly found in *Mycobacterium* spp. and other intracellular pathogens, suggesting exposure to bacterial species that persist within mammalian hosts [22–26] or uptake of environmental species upon feeding. The VF profile shared between *M. arvalis* and *O. aries* is more diverse, including adhesion-related genes (Type IV pili, *E. coli* fimbriae), toxin genes (*CDT*, *Hemolysin*), and secretion systems (T6SS, T7SS, Dot/Icm of a *T4SS*). Many of these factors are associated with Gram-negative enteric pathogens and Gram-positive opportunists, which could suggest interactions with host-associated bacterial communities. For both VF profiles, however, given their frequent occurrence in soil, water, and fecal microbiota, their presence in both voles and livestock does not necessarily indicate recent or direct transmission. Instead, these findings likely reflect the background presence of virulence-associated genes in environmental bacteria that both wildlife and livestock are exposed to [27].

The ARG profiles in *M. arvalis*, particularly the frequent detection of tetracycline and vancomycin resistance genes, also preferentially suggest environmental acquisition rather than direct transfer from livestock. Vancomycin-resistant enterococci have been identified in certain rodent species (*Apodemus sylvaticus*, *Rattus rattus*) [28, 29], though data on their prevalence are limited; specific information regarding the prevalence of vancomycin resistance in *M. arvalis* is currently lacking. In our study, the *M. arvalis* samples showed the highest diversity of antimicrobial resistance gene groups. Durso, Harhay [30] pose the theory, that rodents harbor ARGs and VFs and transmit them to the grazing livestock. Aminoglycoside, tetracycline, and vancomycin resistance genes are commonly found in environmental metagenomes [31–34], implying that *M. arvalis* here could have acquired them rather through food or environmental exposure than by host-to-host transmission.

The limited overlap in ARGs between *M. arvalis* and livestock would suggest that direct transmission events are rare. The presence of a single plasmid-encoded gene in both *M. arvalis* and *B. taurus* hints at the possibility of horizontal gene transfer, whereas the shared genes between *M. arvalis* and *O. aries* - mostly transporters - imply environmental or dietary influence.

*B. taurus* specific ARGs, many of which were plasmid-encoded and commonly found in both Gram-negative and Gram-positive pathogens, indicate a history of gene exchange, potentially influenced by interactions with other livestock or human-associated microbial communities. Similarly, the presence of numerous aminoglycoside resistance genes in *O. aries* highlights possible host-specific selective pressures.

### Limitations and future direction

While our study provides valuable insights into microbial community composition and gene profiles in wildlife and livestock microbiota, several limitations should be acknowledged. First, the fecal sample collection methods differed between livestock and *M. arvalis*: livestock feces were collected off the ground after grazing had ended, whereas the vole samples were obtained directly from dissected guts. This discrepancy may introduce variability in microbial composition and gene detection. Second, the relatively small sample size limited our ability to perform robust statistical analyses, which constrains the generalizability of some findings with respect to the description of the alpha and beta diversity analysis. Third, although the total number of MAGs recovered was modest (58 MAGs across all host species), the completeness and quality of these MAGs permit and support a meaningful interpretation of the community structures.

Including standardized long-term datasets and extensive environmental assessments, which are collected and provided by the DFG Biodiversity Exploratories, enhances comparability across studies, supports longitudinal investigations, and improves the ability to link microbial transmission dynamics to broader ecological patterns, enabling reliable and contextualized conclusions. Expanding this line of research within the Biodiversity Exploratories could advance our understanding of wildlife-livestock interactions, particularly the transmission of microbiota, VF genes, and ARGs.

Additionally, integrating long-read sequencing technologies, such as provided by PacBio or Oxford Nanopore, could improve species identification and genome assembly by generating longer reads, thereby enhancing the resolution of complex genomes, especially for low-abundance species. Although more costly, these technologies can substantially improve data quality, making them a valuable asset for future studies.

## Conclusion

In this study, we analyzed the bacterial community composition, the ARGs, and the VF genes in two livestock species (*Bos taurus* and *Ovis aries*) and in a wild rodent species (*Microtus arvalis*) inhabiting shared grassland plots within the long-term DFG Biodiversity Exploratory “Hainich-Dün.” Our findings revealed host-specific differences in microbial composition. The detection of ARGs in *M. arvalis*, particularly those conferring resistance to tetracycline and vancomycin, along with shared VF profiles between *M. arvalis* and livestock, suggests that environmental acquisition is more likely than direct transmission from the livestock microbiota. These results underscore the importance of distinguishing between true transmission events and shared environmental reservoirs when interpreting metagenomic data. They also highlight the potential influence of land-use practices and habitat connectivity on microbial gene flow, and emphasize the need for future studies to incorporate environmental sampling in order to better understand transmission dynamics and ecological risks. Overall, our study highlights the crucial role of environmental reservoirs in shaping microbial communities across wildlife and livestock.

## Materials and methods

### Sampling strategy, sample Preparation and sequencing

**Fig. 7:**
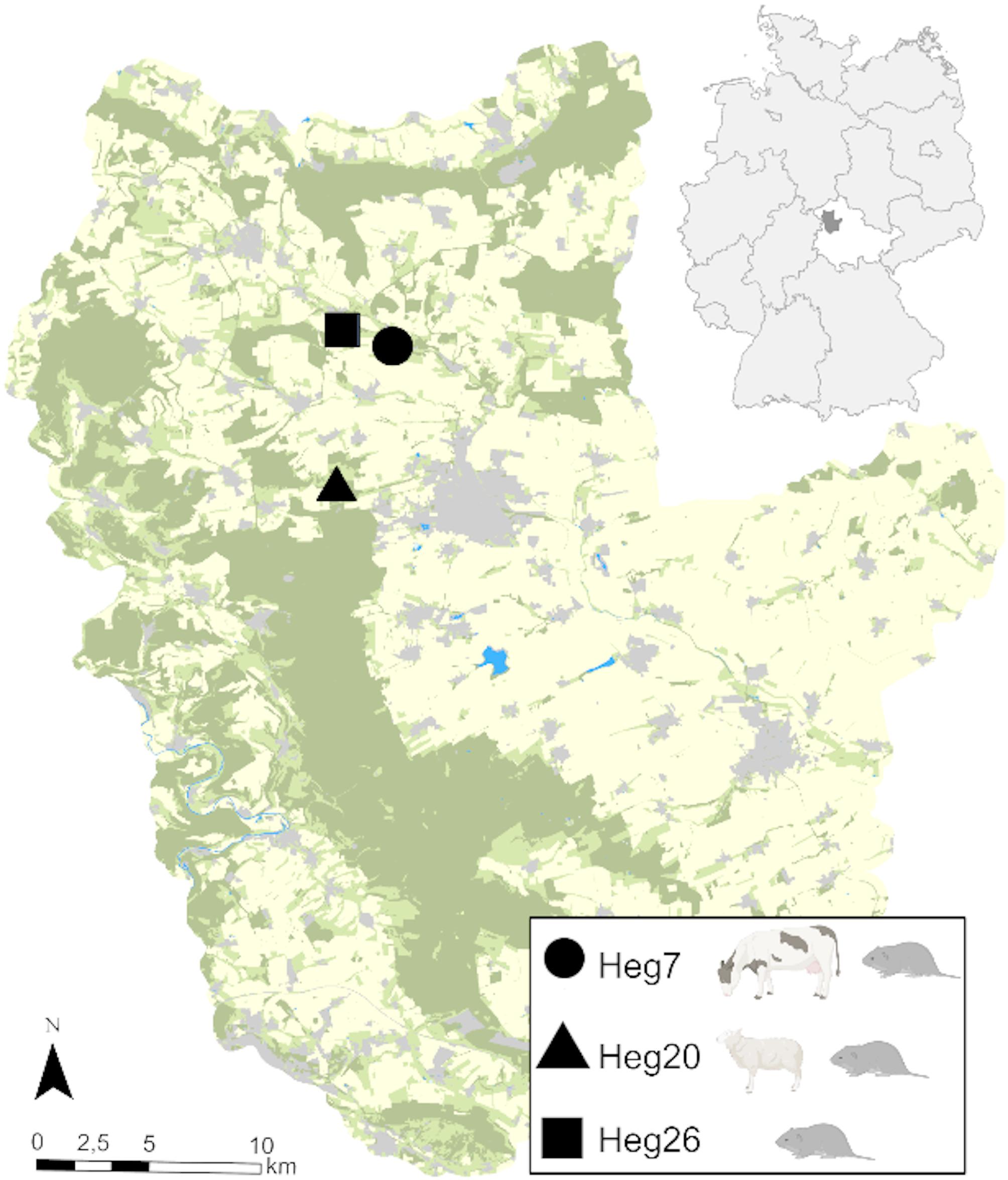
Geographic location of sampling plots

Fecal matter from *Bos taurus* (cattle), *Ovis aries* (sheep), and *Microtus arvalis* (common voles) was collected in September 2020 on three grassland plots (Heg7, Heg20, Heg26) located in the Hainich-Dün area in Thuringia, Germany. The study plots are part of the long-term, large-scale DFG Biodiversity-Exploratories project [35] (Fig. 7), which offers long-term and extensive environmental datasets and parameters. Sampling of rodents took place immediately after the grazing of livestock had ended. Hence, the sampled livestock (*B. taurus* or *O. aries*) and wildlife (*M. arvalis*) inhabited the same plot at the same time during the grazing period. Heg7 (Ma/Bt) was grazed by cattle, therefore, we analysed *B. taurus* feces and *M. arvalis* feces. Heg20 (Ma/Oa) was grazed by sheep, therefore, we analysed *O. aries* feces and *M. arvalis* feces. Heg26 (Ma) acted as a reference plot and only *M. arvalis* feces were analysed, since neither grazing nor organic fertilization had taken place on this plot in 2020.

Feces of *B. taurus* and *O. aries* collected from the grassland plots were pooled and stored at -80 °C. Fecal matter of *M. arvalis* was derived from dissected guts of ten *M. arvalis* individuals, pooled and stored at -80 °C. All procedures involving animals were conducted according to relevant legislation and by permission of the Thuringian State Office of Consumer Protection (permit 22-2684-04-15-105/16).

For analysis, the DNA of the five pooled samples was extracted using the Qiagen QIAamp DNA stool kit according to the IHMS protocol Q [38]. The concentration of the DNA was determined using a NanoDrop™ One/OneC Microvolume UV-Vis Spectrophotometer (Thermofisher, Dreieich, Germany).With these DNA preparations, we conducted amplicon sequencing of five pooled samples. To amplify the V4-5 region of the bacterial 16S rRNA gene, the primer pair 515fF-Y/926R [36] was used. The samples were tagged with barcodes added to the 5’-end of each forward primer in order to be able to multiplex multiple samples in one library. Sixteen robust barcodes were generated using the R-package DNABarcodes version 1.32.0 [37] (barcode length = 9, dist = 5, metric="seqlev”, heuristic="ashlock”). PCR was performed using Q5^®^ High-Fidelity DNA Polymerase (NewEngland Biolabs, Frankfurt/Main, Germany) and the following reaction conditions: 98 °C for 30 s, followed by 30 cycles at 98 °C for 10 s, at 56 °C for 30 s and at 72 °C for 60 s, and then one cycle at 72 °C for 2 min. Before library construction, a clean-up step was performed using the NucleoSpin^®^ Gel and PCR Clean-up kit (Macherey & Nagel, Düren, Germany).

For metagenome sequencing, aliquots of the same DNA preparations were used to construct six DNA sequencing libraries (five metagenomic libraries and one 16 S multiplexed library) according to the manufacturer´s instructions using the NEBNext Ultra II DNA Library Prep Kit for Illumina (New England Biolabs) and NEBNext Multiplex Oligos for Illumina 96 Unique Dual Index Primer Pairs Set1 (New England Biolabs). Paired-end (2 × 250 bp) sequencing was conducted by IMGM Laboratories (Martinsried, Germany) on a NovaSeq 6000 with the SP 500 v1.5 Kit (Illumina, Berlin, Germany) and the NovaSeq XP 2-Lane Kit v1.5 (Illumina) [39].

Data from Kauer, Imholt [14], in which *M. arvalis* samples from the same plots were analysed individually via 16 S rRNA gene fragment sequencing, were used to compare the newly generated results of the *M. arvalis* pooled samples to *M. arvalis* samples processed and sequenced individually by the previous study Kauer, Imholt [14]. We used the data of two individuals from each plot (Heg7, Heg20 and Heg26), that had also been caught in September 2020; sample processing was identical to the pooled samples with the exception that DNA extraction, amplification and sequencing were conducted separately for each sample, see also Kauer, Imholt [14] for details.

Raw sequence reads were submitted to the DNA database EMBL EBI ENA database [39] with the accession number PRJEB86517.

### Read data processing and statistical analysis

For analyzing the V4-5 region of the bacterial 16 S rRNA gene, we generated an Amplicon Sequence Variants (ASV) table using the standard DADA2 denoising pipeline (Callahan et al., 2016), including quality filtering and removal of chimeric sequences within QIIME2 vers. 2023.2 [40]. We applied the feature-classifier fit-classifier-naive-bayes function [41] in QIIME2 to build self-trained classifiers, trained on preformatted reference data [42] from the SILVA database derived from the Silva database [43]. Lastly, we conducted prevalence filtering (0.05 = 5%) using metagMisc version 0.5.0 [44] and rarefaction (4.210 reads).

Bacterial taxonomic names were assigned by the taxonomic classification tool used and were not adjusted, meaning nomenclature reflects the tool’s output. All analysis in R was conducted using version 4.3.2 [45]. We imported abundance tables as a phyloseq object, and phyloseq vers. 1.46.0 [46] was used for analysis and visualization. The Shannon index was used to assess alpha diversity, as it accounts for both species richness and relative abundance to provide a comprehensive measure of community diversity. We used Kneaddata vers. 0.12.0 (https://github.com/biobakery/kneaddata) with a custom database for quality control and filtering with the default settings. The custom database is based on the reference sequences, derived from NCBI of the host organisms (*B. taurus* = GCF_000003055.6, *O. aries* = GCF_000298735.2, *M. arvalis* = GCA_007455615.1). Fastqc [47] and Multiqc [48] were used for visualization of the quality-controlled reads.

To generate metagenome-assembled genomes (MAGs), Megahit vers. 1.1.1 [49] was used for the *de novo* assembly of metagenomic reads. To generate bins, we utilized Metabat vers. 2:2.15 [50] with the minimum size of a contig for binning = 1500. Bins were dereplicated with dRep vers. 3.4.0 [51]. CheckM vers. 1.2.1 [52] was used for quality control of the MAGS obtained using the parameters “more than 80% completeness” and “less than 5% contamination”. To assign taxonomy to the MAGs, we used GTDB-tk vers. 2.1.1 [53] and accessed the release 09-RS220 of the GTDB database [54]. A phylogenetic tree was built with FastTree vers. 2.1.11-2 [55] using the alignment from GTDB-tk, and visualized with iTOL [56]. Additionally, taxonomic classification was assigned to reads using Kaiju vers. 1.9.2 [57].

To estimate the abundance and diversity of ARG genes and virulence factors, quality-controlled filtered reads were mapped against the AMRFinderPlus database [58] and the Virulence Factor DataBase, a.k.a. VFdb [59] using the k-mer alignment (kma) software. To normalize reads and create proportions of ARG and of VF reads per bacterial cells in each sample, MicrobeCensus vers. 1.1.0 [60] was used with default settings.

## Electronic supplementary material

Below is the link to the electronic supplementary material.

Supplementary Material 1

## Data Availability

The raw sequence reads generated during this study were submitted to the DNA database EMBL EBI ENA with the accession number PRJEB86517. The metagenome-assembled genomies are publically available via the DOI: 10.6084/m9.figshare.28714991. Interactive iTOL files are publically available via the DOI: 10.6084/m9.figshare.28714991. Interactive iTOL files are publically available via the DOI: 10.6084/m9.figshare.28640609.v1. This work is based on data elaborated by the LabiRo project of the Biodiversity Exploratories program (DFG Priority Program 1374). The dataset is publicly available in the Biodiversity Exploratories Information System (http://doi.org/10.17616/R32P9Q) under the Id 32082 and title “Metagenomic analysis of Bos taurus, Ovis aries and Microtus arvalis fecal pool samples“.
