# Correction: Microbial exchange at the wildlife-livestock interface: insights into microbial composition, antimicrobial resistance and virulence factor gene dynamics in grassland ecosystems

**DOI:** 10.1186/s42523-025-00458-0

**Published:** 2025-09-01

**Authors:** Lea Kauer, Panagiotis Sapountzis, Christian Imholt, Christian Berens, Ralph Kuehn

**Affiliations:** 1 Molecular Zoology, Department of Zoology, TUM School of Life Sciences, Technical University of Munich, Munich, Germany; 2 Medis 0454, 63122, INRAE-UCA, Centre INRAE Auvergne-Rhône-Alpes, Site de Theix, Saint-Genès-Champanelle, 63122 France; 3 Federal Research Centre for Cultivated Plants, Institute for Epidemiology and Pathogen Diagnostics, Rodent Research, Julius Kühn-Institute, Münster, Germany; 4 Friedrich-Loeffler-Institut, Institute of Molecular Pathogenesis, Jena, Germany; 5 Department of Fish, Wildlife and Conservation Ecology, New Mexico State University, Las Cruces, NM USA

**Correction: anim microbiome 7**,** 84 (2025).**

10.1186/s42523-025-00448-2

Following publication of the original article [1], the authors reported an error in the results section “Antimicrobial resistance genes” where the second sentence contains two citations “38” and “26”.

These should not be citations, but rather numbers (ARG counts in the respective sample) and should read as “(N = 38)” and “(N = 26)”.

The correct sentence should then read as follows:

“The *O. aries* sample held the most ARGs (N = 38), followed by the *B. taurus* sample (N = 26) and the *M. arvalis* samples from Heg20 (Ma/Oa): 26, Heg7 (Ma/Bt): 21, and Heg26 (Ma): 15.”

