# Supplementary material for: Microbial exchange at the wildlife-livestock interface: insights into microbial composition, antimicrobial resistance and virulence factor gene dynamics in grassland ecosystems
Source: Anim Microbiome. 2025 Aug 6;7:84. doi: 10.1186/s42523-025-00448-2 (PMC12330063; doi:10.1186/s42523-025-00448-2)
Supplement: Supplementary file 1 — Supplementary Material 1 [file 42523_2025_448_MOESM1_ESM.docx]

**Table S1**, Top five most abundant bacterial groups in the individual M. arvalis samples, based on 16S rRNA sequencing, for all three plots separately

| **Host species and sample ID** | **Subphylum taxonomic unit** | **Abundance [%]** |
| --- | --- | --- |
| *M. arvalis*  - individual samples  Heg7 | *Muribaculaceae* | 25.2 |
|  | *Chloroplast* | 17.5 |
|  | *Christensenellaceae* | 15.4 |
|  | *Desulfovibrionaceae* | 10.7 |
|  | *Streptococcaceae* | 5.7 |
| *M. arvalis*  - individual samples  Heg20 | *Muribaculaceae* | 24.4 |
|  | *Desulfovibrionaceae* | 19.1 |
|  | *Christensenellaceae* | 14.8 |
|  | *Chloroplast* | 9.1 |
|  | *Lachnospiraceae* | 6.6 |
| *M. arvalis*  - individual samples  Heg26 | *Muribaculaceae* | 23.9 |
|  | *Desulfovibrionaceae* | 11.2 |
|  | *Christensenellaceae* | 10.3 |
|  | *Lactobacillaceae* | 9.3 |
|  | *Chloroplast* | 8.8 |

**Table S2**, Top five most abundant bacterial groups in the pooled M. arvalis samples, based on 16S rRNA sequencing, for all three plots separately

| **Host species and sample ID** | **Subphylum taxonomic unit** | **Abundance [%]** |
| --- | --- | --- |
| *M. arvalis*  - pooled samples  Heg7 | *Christensenellaceae* | 44.4 |
|  | *Desulfovibrionaceae* | 18.8 |
|  | *Muribaculaceae* | 10.3 |
|  | *Lachnospiraceae* | 7.1 |
|  | *Erysipelotrichaceae* | 5.5 |
| *M. arvalis*  - pooled samples  Heg20 | *Erysipelotrichaceae* | 23.2 |
|  | *Muribaculaceae* | 22.6 |
|  | *Desulfovibrionaceae* | 20.9 |
|  | *Lachnospiraceae* | 8.6 |
|  | *Christensenellaceae* | 8.1 |
| *M. arvalis*  - pooled samples  Heg26 | *Muribaculaceae* | 25.5 |
|  | *Desulfovibrionaceae* | 19.4 |
|  | *Christensenellaceae* | 14.5 |
|  | *Lachnospiraceae* | 13.4 |
|  | *Lactobacillaceae* | 8.0 |

*
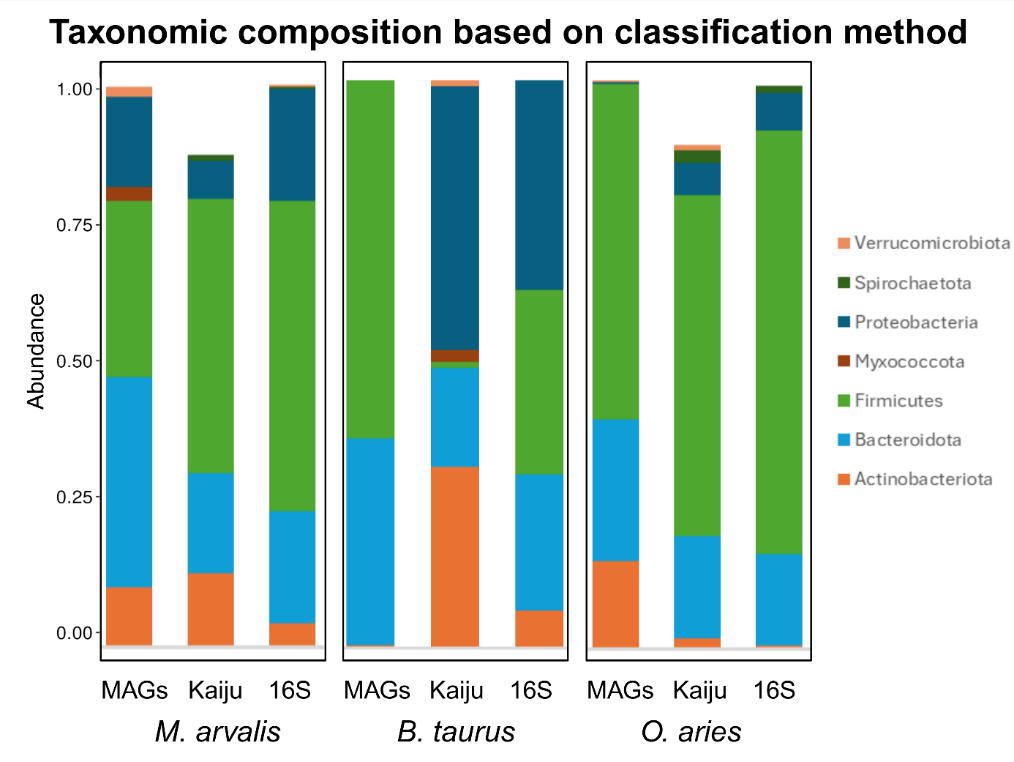
*

**Figure 1**, Taxonomic composition of the M. arvalis, B. taurus and O. aries samples based on the classification used for bacterial phyla
